# Visual working memory phenomena based on categorical tasks replicate using a continuous measure: A simple interpretation and some methodological considerations

**DOI:** 10.3758/s13414-023-02656-x

**Published:** 2023-02-08

**Authors:** Yanmei Hu, Richard J. Allen, Alan D. Baddeley, Graham J. Hitch

**Affiliations:** 1 Northeast Normal University, Changchun, China; 2 School of Psychology, University of Leeds, Leeds, UK; 3 Department of Psychology, University of York, York, UK

**Keywords:** Visual working memory, Attention

## Abstract

An increasingly popular method for investigating visuospatial working memory assumes stored features of objects such as color and orientation vary along continua subject to internal noise. It adapts the stimulus adjustment procedure from perceptual psychophysics to assess the precision with which stored features are represented in memory. This contrasts with methods using discrete, categorical measures of feature retention. The current study examined the replicability of some phenomena documented using conventional methodology when assessed using a continuous measure of feature recall. These concern memory for a short series of objects and include effects of recency, prioritizing an individual object, and presenting an irrelevant additional object after the last item (a poststimulus ‘suffix’). In two experiments we find broadly similar results using a continuous measure of color-orientation binding to those obtained previously using categorical measures, with small differences we regard as minor. We interpret the convergence between methods in terms of a simple analogy between categorical memory and categorical perception whereby categorical retrieval involves the application of a discrete criterion to an underlying continuum of stored feature information. We conclude by discussing some of the advantages and limitations of continuous and categorical measures of retention.

There has, in recent years, been an increasing concern with the robustness of published evidence about psychological phenomena, and the extent to which they are replicable when performed in other laboratories (e.g., Elliott et al., 2021; Open Science Collaboration, 2015). Some of these failures to replicate may result from subtle differences in methodology or in participants, making it important that such variables are carefully specified and controlled across studies. One danger, however, is a potential for this to narrow the range of investigation, resulting in an increasingly paradigm-based literature that may have little generality within or applicability beyond the laboratory. This is a particular danger when an area is approached from two different traditions, each having different strengths based on different methods, which can potentially lead to two separate noninteracting literatures but, if combined, can strengthen the overall theory.

We suggest that visual working memory is a good example of a field with two complementary but different approaches. One is earlier work principally concerned with its role in visual imagery, complex processing and long-term memory (Baddeley & Lieberman, 1980; Logie, 1986; Shepard & Metzler, 1971), although a notable exception to this was the work of Phillips (Phillips, 1974; Phillips & Baddeley, 1971; Phillips & Christie, 1977) that was a direct forerunner of the currently dominant approach to visual working memory. This more recent approach has stemmed from research on visual attention and focuses on a more detailed understanding of the earlier stages of visual memory (e.g., Bays et al., 2009; Kahneman et al., 1992; Luck & Vogel, 1997; Wheeler & Treisman, 2002; Zhang & Luck, 2008; for a review see Bays et al., 2022). Our own work in recent years has attempted to bridge the two fields by applying methods that were originally developed for verbal material to their visual equivalent (for reviews, see Baddeley et al., 2011a, 2021, and Hitch et al., 2020). As in the case of most verbal experiments we have tended to use categorizable material such as nameable colors or shapes, whereas a good deal of recent work on visual working memory has used continuous measures based on response precision and has allowed more detailed models to be proposed and tested (Bays & Husain, 2008; Bays et al., 2009; Ricker et al., 2022; Wilken & Ma, 2004; Zhang & Luck, 2008). This contrast leads to the motivation and aims of the present study. Our basic concern is not with testing such models but rather with asking whether categorical methods (e.g., item recall and recognition) taken from verbal paradigms are sufficiently robust as to replicate using continuous response methods. We conclude with a brief discussion of the strengths and weaknesses of continuous and categorical methods.

There certainly are examples of basic phenomena detectable by either method. One is the well-known recency effect in immediate recall whereby memory for individual visual items in a sequence tracks their recency of presentation. We have consistently observed a recency effect when memory for a series of colored shapes is assessed by categorical measures such as the ability to name the original color of an achromatic test shape (Allen et al., 2006, 2014a, 2017; Atkinson et al., 2018; Atkinson et al., 2019; Berry et al., 2018; Hitch et al., 2018). In experiments using a continuous measure, Gorgoraptis et al. (2011) had participants view a series of oriented colored bars and then rotate a colored test bar to match its previous value. There was a clear recency effect in that the precision of these responses was highest for the final item and declined progressively over earlier serial positions.

Another set of phenomena detectable by either method concerns the effects of prioritizing items within a series or array. We have reported several investigations in which we rewarded categorical recall of items with different numbers of points according to their serial position during presentation (Allen et al., 2021; Allen & Ueno, 2018; Atkinson et al., 2018; Atkinson et al., 2019; Hitch et al., 2018; Hu et al., 2014; see Allen, 2020, and Hitch et al., 2020, for reviews). The main finding was enhanced recall of high-priority items combined with a cost to the recall of low-priority items, such that the overall amount of information retained remained the same. This trade-off is theoretically interesting as it suggests prioritization alters the allocation of a limited pool of attentional resources. Using their continuous adjustment method, Gorgoraptis et al. (2011) manipulated prioritization by varying the likelihood of probing memory for a particular color of bar. The orientation of the bar more likely to be tested was recalled with greater precision and there was a corresponding loss of precision in recalling other items. Stochastic modelling showed a reduction in responses based on the orientation of an incorrect bar, consistent with stronger feature binding for prioritized items. Interestingly, a reexamination of our own data indicates a corresponding effect on feature binding in categorical recall. More specifically, prioritizing an item tended to reduce the probability of incorrectly recalling a feature from a different item in the series (see Table 1, Experiments 2 and 3 in Hu et al., 2014).

Another observation detectable by both methods occurs when multiple items are prioritized differentially at the same time. In categorical recall this results in graded effects across items reflecting their levels of priority (Allen & Ueno, 2018; Hitch et al., 2018). Using a continuous measure of feature recall, Klyszejko et al. (2014) found similarly that when items were given different priorities, the precision with which they were recalled covaried with their rank order of priority.

Hence, there is no doubt that some results can be detected by either method. However, the fact that some measures apply to both is not a convincing general argument. A good example comes from attempts to study visual working memory in patient Jon, a developmental amnesic patient with greatly impaired recall but preserved repetition following perinatal anoxia (Baddeley et al., 2001; Vargha-Khadem et al., 1997), where his excellent visual working memory argues against the proposal of a central role of the hippocampus in working memory (Allen et al., 2014b; Baddeley et al., 2010, 2011b). Our studies always used a limited set of colours or shapes, leading open the suggestion that Jon’s problem might be with the precision of responding (Ekstrom & Yonelinas, 2020; Yonelinas, 2013). Although this proved not to be the case in this instance (Allen et al., 2022), there is evidently potential value to be gained from looking at major phenomena using both methodologies. This has been nicely illustrated by studies of visual working memory for color showing that performance reflects a combination of categorical and continuous information (Bae et al., 2015; Hardman et al., 2017), with the precise nature of the representation appearing to vary depending on the task that is implemented (Ricker et al., 2022).

The aim of the present study is to investigate whether three further characteristics of visual working memory identified using categorical measures are sufficiently robust as to be readily detectable using a continuous measure. The first involves another look at the effects of prioritization. Although as we have noted Gorgoraptis et al. (2011) and Klyszejko et al. (2014) reported effects on continuous measures that parallel our findings using categorical recall (Allen et al., 2021; Allen & Ueno, 2018; Atkinson et al., 2018; Atkinson et al., 2019; Hitch et al., 2018; Hu et al., 2014), they manipulated prioritization by increasing the probability of testing items of a particular color, whereas we used instructions assigning importance to items according to their serial position. The former may have a learned, relatively automatic effect (Atkinson et al., 2018), whereas the latter is more likely to be mediated by top-down attentional control processes (Hu et al., 2016). Given the potential importance of this distinction, we were interested to see whether the effects on continuous recall of an item’s probability of being tested can be replicated when its priority is instead determined by instructions.

The second characteristic concerns the effect of a visual distractor item presented immediately after the final item in a to-be-remembered series. The typical effect of such a “stimulus suffix” on visual working memory is poorer categorical recall of the last one or two items but no effect on earlier items (Hu et al., 2014). We interpret this as suggesting the suffix displaces the most recently presented items from the focus of attention, which has limited capacity (Hitch et al., 2018). If visual information in the focus of attention is represented both categorically and continuously, we would expect to see similar effects of a stimulus suffix on either form of measure. However, to the extent that the focus of attention is a separate state within working memory with distinct characteristics (Cowan, 1995; Cowan et al., 2021; Hitch et al., 2018; Hitch et al., 2020), different results would not be entirely surprising.

The third and final characteristic of visual working memory we investigate here concerns the way prioritization instructions alter the effect of a suffix. In previous work we have found that prioritizing the first item in a series not only boosts its recall but also renders it susceptible to interference from a suffix, in contrast with the absence of such interference when the first item is not prioritized (Hu et al., 2014). We interpreted these results as suggesting that instructions to prioritize an item increase the probability of it being in the focus of attention at the time of recall, presumably by biasing the schedule of attentional refreshing (Barrouillet & Camos, 2021). As above, replication of these effects using a continuous measure of visual working memory may have implications for our understanding of the way visual information is represented inside and outside the focus of attention.

To investigate the generality of this set of phenomena, we report two experiments each employing a continuous response task. This investigated the recall of one item from a series of three rotated colored bars, following the methodology of Gorgoraptis et al. (2011). Following a brief retention interval one of the bars was redisplayed in a different random orientation and the participant asked to rotate it back to its original state. Recall is measured on a continuous scale by the difference between the remembered orientation and the original. We compared performance in the absence of prioritization instructions with performance under instructions to prioritize one of the items by associating its accuracy with a bigger reward. Without prioritization instructions all items were equally important, and we expected to replicate the through-sequence recency effect observed by Gorgoraptis et al. (2011). If, further, the prioritization effects we obtained previously using categorical recall generalize, we would also expect to see reduced error and higher precision in recalling a high-priority item offset by the reverse on low-priority items. Such a trade-off would be consistent with prioritization altering the allocation of a limited pool of resources to offset the rapid forgetting of continuous information as we have proposed for categorical information (Hitch et al., 2020).

Our second experiment moved on to reexamine effects of prioritization and serial position in the presence of a poststimulus suffix. The suffix was a rotated bar with a color drawn from the same pool as to-be-remembered items but not among them on that trial. Participants were instructed to ignore any suffix they saw and, in blocks of trials when they were so instructed, to prioritize the first item in the sequence. In addition to seeking to provide a further replication of the effects of serial position and prioritization, we were interested in whether a suffix would have parallel effects to those seen in categorical recall. If so, presentation of a suffix would increase error and reduce precision for the most recent item regardless of prioritization instructions, but only do the same for the first item when it is prioritized. This would be consistent with our evidence from categorical recall and the suggestion that prioritization alters the schedule of attentional refreshing whereby categorical and continuous information about individual items are cycled through the focus of attention.

## Experiment 1

To reiterate briefly, our first experiment set out to replicate our previous findings on immediate memory for a series of visual stimuli when retention is assessed in terms of the precision of feature recall rather than a categorical measure (Allen, 2020, and Hitch et al., 2020, for reviews). Full replication would consist of a through-sequence recency effect, enhanced retention of high-priority stimuli and reduced retention of low priority stimuli, with a trade-off between the two resulting in no change in overall performance. Such outcome would also replicate the findings of Gorgoraptis et al. (2011), but with prioritization based on assigning rewards as a function of an item’s serial position rather than the probability of testing an item as a function of its color.

Only one study we know of has looked at the effects of nonpredictive prioritization based on rewards using a continuous measure of retention (Atkinson et al., 2022). This used simultaneous presentation of an array of colored shapes followed, after a brief retention interval, by probed recall of the color of a randomly chosen shape using a continuous color wheel. As expected, color was recalled with greater precision for an item assigned high priority than for items assigned low priority, and there was no benefit of prioritization on overall performance relative to a condition in which items had equal priority. Atkinson et al. (2022) also explored whether prioritization effects are mediated by attentional refreshing by on some trials postcueing participants to ‘think of’ one of the items during the retention interval. They found postcueing enhanced the retention of low or equal value items but had no effect on high value items, interpreting this as supporting the hypothesis that prioritization effects are at least partially mediated by attentional refreshing, given the assumption that high-value items would be refreshed regardless of whether they are cued. However, no study has yet explored the effect of value-based prioritization in the context of sequential presentation to allow examination of prioritization and serial position effects in conjunction.

### Method

#### Participants

The required sample size was estimated using G*Power (Faul et al., 2007). According to our previous study using categorical measures (Hitch et al., 2018), which found a moderate effect size (Cohen’s *d* = .576) of prioritization, a power analysis showed that 42 participants would be sufficient to achieve a power of 0.95 at alpha level of 0.05. Fifty-four students (aged 18–32 years, mean age 22 years, 41 females, 13 males) from the University of York and the Northeast Normal University of China were tested individually and were paid or given course credit for participation. All participants reported having normal color vision.

#### Materials

Experiment 1 was run on a Pentium PC with a 21-in. screen, using E-Prime (Version 2.0). Stimuli were presented against a white background and viewed from approximately 50 cm. Study items were colored bars (approximately 0.5° × 3°) with random orientations except 90° and 180°, and one of eight possible colors (red, blue, yellow, green, sky blue, purple, gray, and black). No orientation or color could appear more than once among study items in each trial. The test probe was a colored bar corresponding to one of the study items but with a random orientation.

#### Design and procedure

Experiment 1 comprised six blocks: (1) a pretest practice block with one item, (2) two baseline (no-priority) blocks, one following the practice and one at the end of the session, and (3) three priority condition blocks implemented between the baselines. Each block consisted of nine practice trials and 30 experimental trials.

**Fig. 1:**
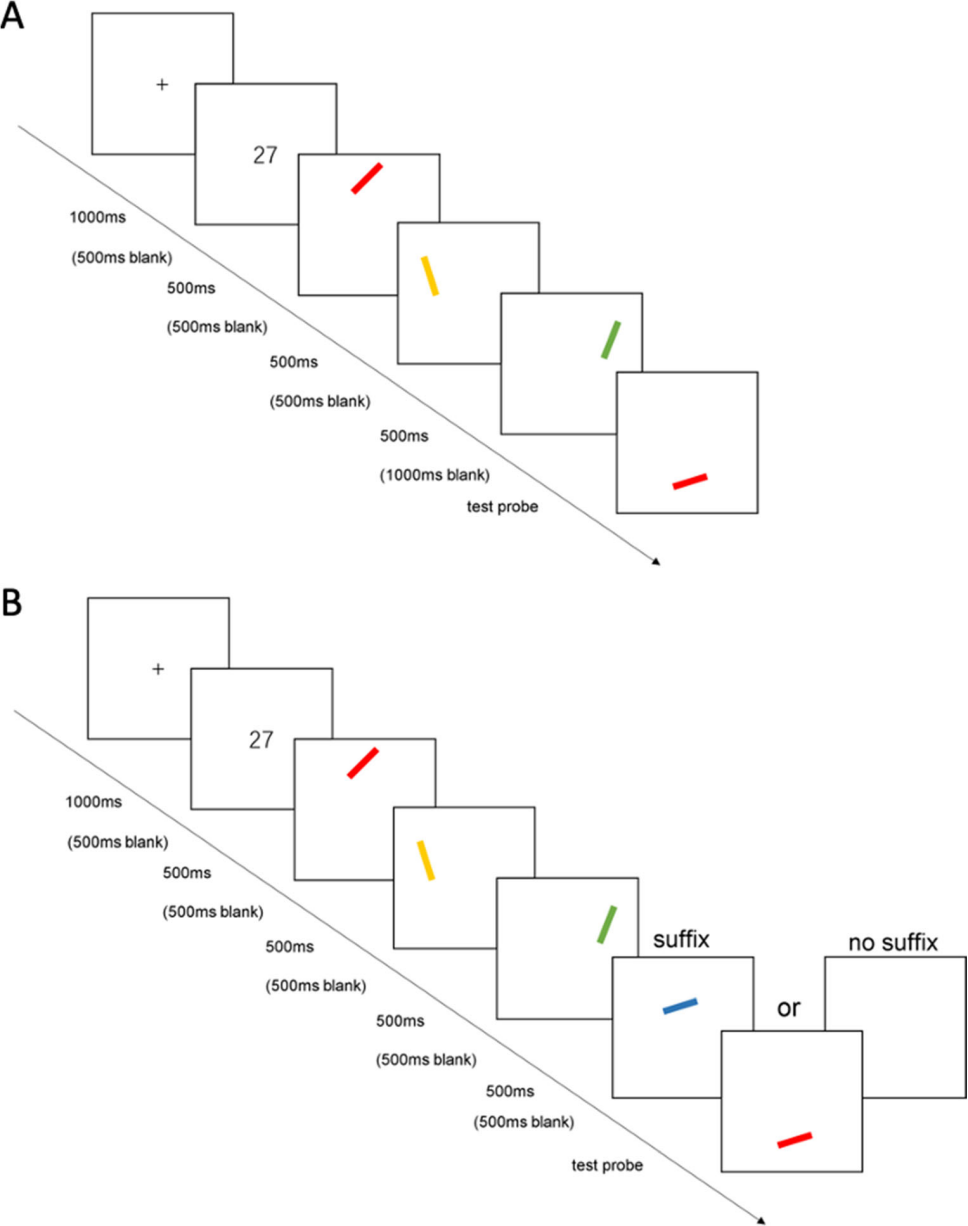
Schematic outline of procedures used. **A** Experiment 1. **B** Experiment 2. (Colour figure online)

Figure 1 illustrates the procedure. Each trial began with a 500-ms warning cross followed by a 500-ms blank screen. Next, a two-digit number chosen randomly from the range 10–99 was shown for 1,000 ms. Participants were required to repeat the number aloud at a speed of 2–4 times per second from its onset until the onset of the test probe. Concurrent articulation was required to discourage the use of verbal recoding and subvocal rehearsal. Next, 500 ms after the offset of the number, study items were presented sequentially for 500 ms each separated by 500-ms blank intervals. The three study items were presented in a random spatiotemporal sequence, assigned to the 12, 4, and 8 o’clock positions of an invisible circle (approximately 9° in diameter, centered 4° above the center of the screen). The final item was followed by a 1,000-ms blank interval, and then the test probe was presented approximately 5° below the center of the screen. Participants were required to recall the orientation of the study item that had the same color as the probe. They did this by selecting a location on the screen by clicking the mouse. A second click rotated the probe clockwise or anticlockwise to point to the position of the mouse. The initial one-item practice trials were the same as the above, but only involved presentation of one stimulus (at screen centre), followed by the test probe.

In the baseline no-priority condition participants were told they would receive two points for correct recall no matter which item was probed. In the prioritization condition blocks the procedure was identical except that different numbers of points were allocated to study items as a function of their serial position. In the SP1-Priority condition, participants were told they would receive four points for correct recall of the first item and one point for each of the others; in the SP2-Priority condition, four points were given for correct recall of the second item and one point each for the others; and in the SP3-Priority condition, four points were given for correct recall of the last item and one point each for the others.

The order of administering the three prioritization conditions was counterbalanced across participants. Across the session, there were 150 trials in total (excluding practice trials), with each serial position probed 50 times. There were 60 baseline no-priority trials, 30 trials on which a high-value item was probed, and 60 trials on which a low-value item was probed (i.e., when the higher value in the sequence was assigned to a nontested item).

#### Data analysis

Outcome variables were generated using the Mixtur package (Grange & Moore, 2022) in R (see also, Allen et al., 2022). We focus on the model-free summary statistics (absolute error and precision) produced by this approach. Absolute error represents the circular mean of the absolute deviation between the response value and the true target value (limited to the 0–180-degree range), with values closer to zero representing greater accuracy of response. Precision is calculated as the reciprocal of the standard deviation for circular data, minus the value expected by chance (Bays et al., 2009; Grange & Moore, 2022).

Outcomes were analysed in JASP (Version 0.16.3), using a combination of frequentist and Bayesian analyses of variance (ANOVAs) and *t* tests (e.g., Allen et al., 2021; Atkinson et al., 2018; Atkinson et al., 2021; Atkinson et al., 2022). Greenhouse–Geisser corrections were applied where appropriate. Bayes factors (BF) are reported (using default priors) as a continuous estimation of the strength of evidence for the data under the null and alternative hypotheses (e.g., Dienes & Mclatchie, 2018). For ANOVAs, these correspond to BF_incl_ (i.e., the strength of evidence for the inclusion of each factor and interaction in the model). For *t* tests, BF_10_ are reported, indicating evidence for the presence of an effect. In each case, BF < 1 indicates support for the null hypothesis, and BF > 1 support for the alternative hypothesis. We used the standard classification scheme in which BF 1–3 equates to anecdotal evidence, BF 3–10 as moderate evidence, and BF >10 as strong evidence (Jeffreys, 1961; Lee & Wagenmakers, 2013).

### Results

We first compared the two no-priority trial blocks to establish whether performance changed across the experimental session. There was no significant difference between the two blocks for absolute error (Block 1 = .66, *SE* = .03; Block 2 = .65, *SE* = .04), *t*(53) = .50, *p* = .62, *d* = .07, BF = .17, or precision (Block 1 = .67, *SE* = .05; Block 2 = .74, *SE* = .06), *t*(53) = 1.40, *p* = .17, *d* = .19, BF = .37. Thus, response accuracy did not appear to change across the session. All subsequent analysis combines the data from these two no-priority blocks.

**Fig. 2:**
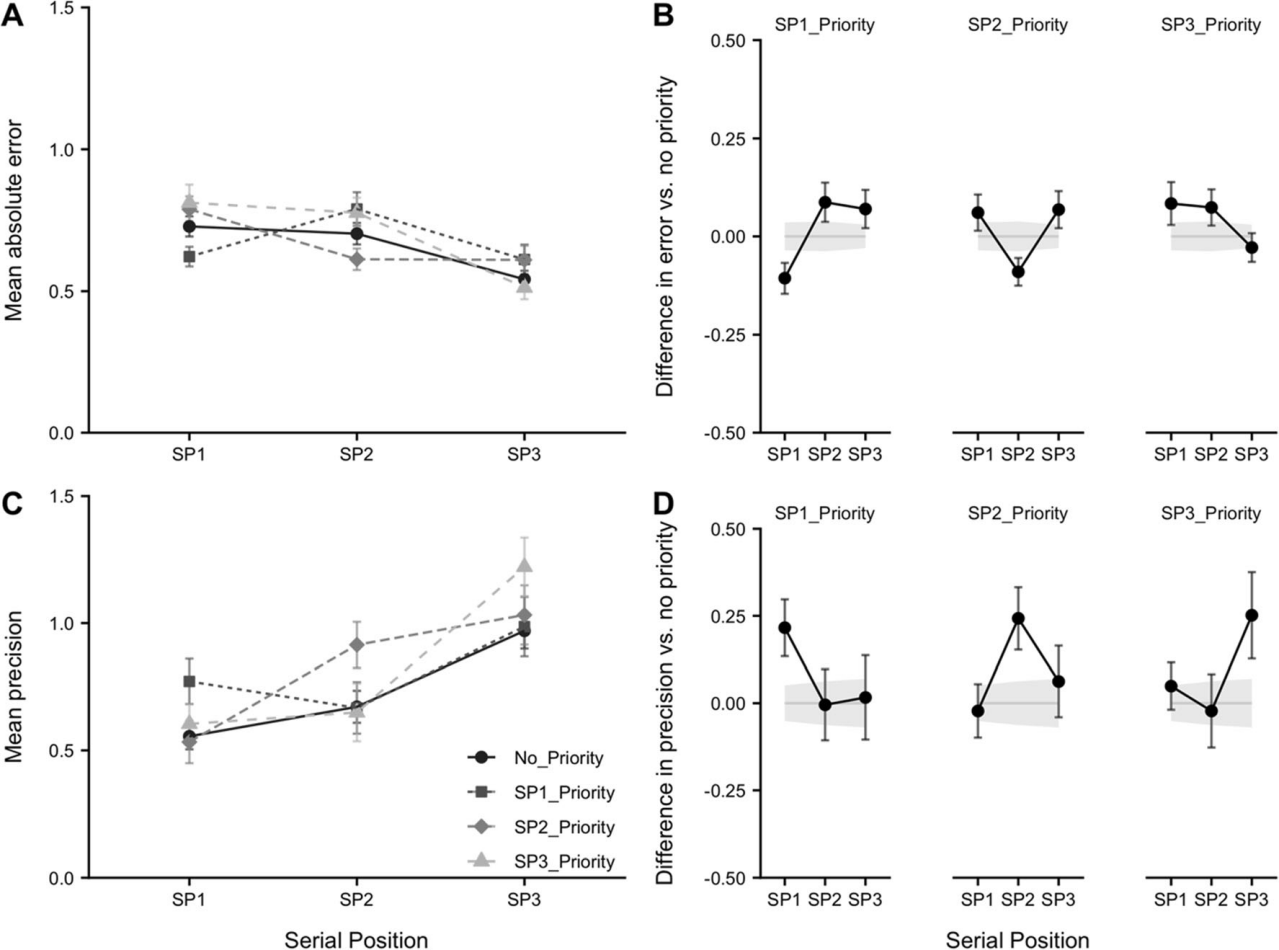
Effects of serial position and prioritization conditions. **A** Mean absolute error (radians). **B** Difference in error relative to no-priority baseline. **C** Mean precision. **D** Difference in precision relative to no-priority baseline. Error bars show standard error (*SE*). Shading in panels *B* and *D* represents *SE* in the no-priority condition

Mean absolute error and precision are displayed in Fig. 2A and 2C, respectively, as a function of priority condition and serial position. We also provide difference scores comparing each priority condition against the no-priority baseline trials (Fig. 2B and D).

A 4 × 3 (priority condition by serial position) repeated-measures ANOVA on each performance measure indicated no significant effect of priority condition: error, *F*(3, 159) = .86, *p* = .47, η_p_^2^ = .02, BF = .02; precision, *F*(2.58, 136.80) = 1.12, *p* = .34, η_p_^2^ = .02, BF = .02. There was an effect of serial position: error, *F*(2, 106) = 34.47, *p* < .001, η_p_^2^ = .39, BF > 1,000; precision, *F*(2, 106) = 31.61, *p* < .001, η_p_^2^ = .37, BF >1,000, with Holm-corrected comparisons indicating reduced error and improved precision at Serial Position 3, compared with Position 1 (*p* < .001, *d* > .63, BF > 1,000), and Position 2 (*p* < .001, *d* > .44, BF > 1,000). Positions 1 and 2 did not differ (*p* > .05, *d* < .17, BF < 1).

The interaction between priority condition and serial position was significant: error, *F*(2.65, 140.55) = 5.27, *p* = .003, η_p_^2^ = .09, BF > 1,000; precision, *F*(4.72, 250.03) = 2.83, *p* = .019, η_p_^2^ = .05, BF = 1.77. The key comparison in each case was between the prioritized serial position and the corresponding position in the no-priority condition. This difference was significant at Serial Position 1 (error, *t* = 2.74, *p* = .008, *d* = .37, BF = 4.22; precision, *t* = 2.65, *p* = .010, *d* = .36, BF = 3.51) and Serial Position 2 (error, *t* = 2.56, *p* = .013, *d* = .35, BF = 2.87; precision, *t* = 2.72, *p* = .009, *d* = .37, BF = 4.03), but there was little support for a difference at Serial Position 3, (error, *t* = 78, *p* = .441, *d* = .11, BF = .20; precision, *t* = 2.03, *p* = .047, *d* = .28, BF = .99).

**Fig. 3:**
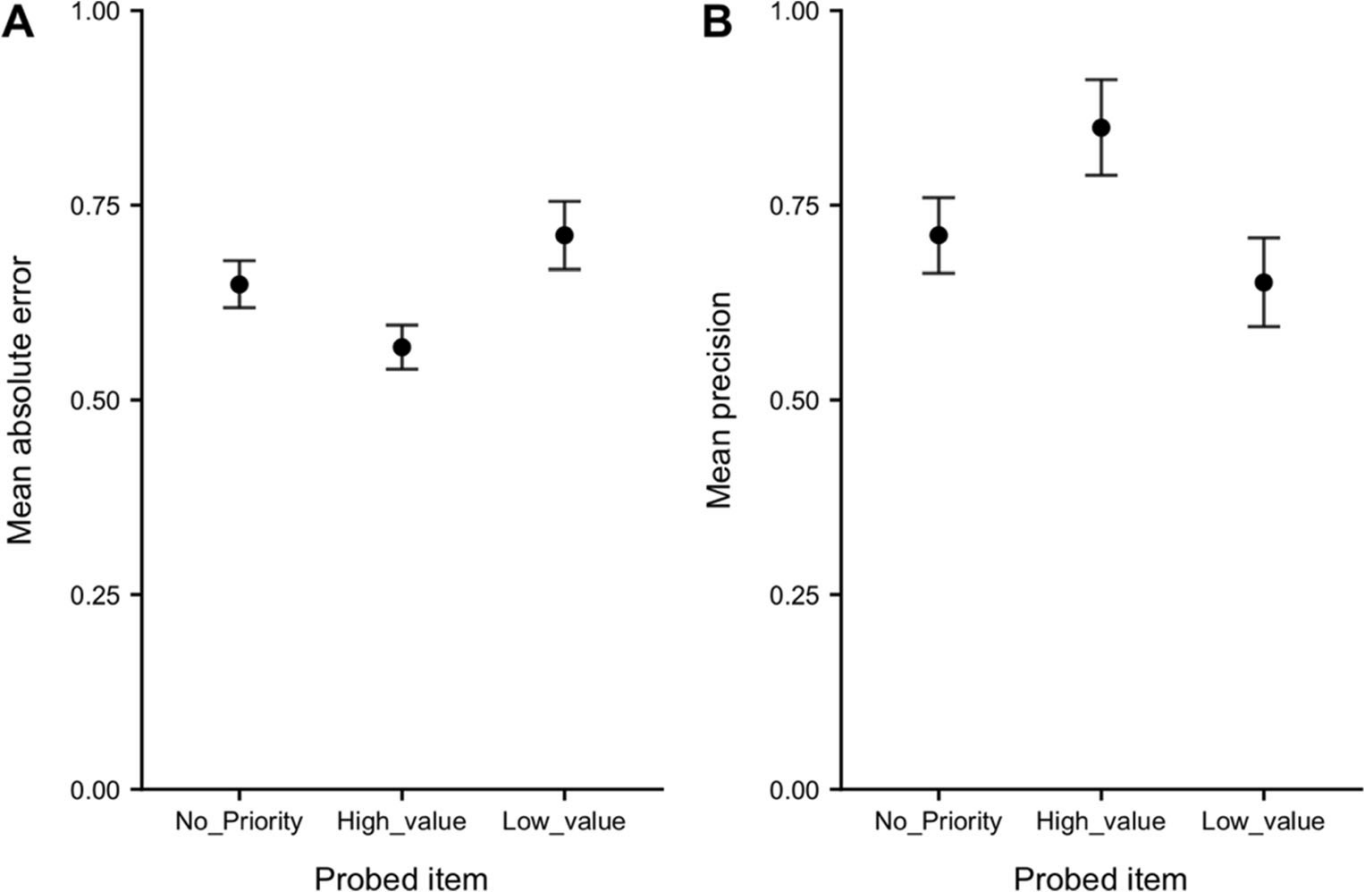
Absolute error (**A**) and precision (**B**) by type of probed item. Error bars show *SE*

As high value was allocated to each serial position across the priority conditions, it was also possible to contrast performance as a function of type of item probed (i.e., no-priority, high priority, low priority). Figure 3 shows the results. A one-way repeated-measures ANOVA on each measure of performance indicated a significant effect of probe type: error, *F*(1.30, 69.03) = 7.98, *p* = .003, η_p_^2^ = .13, BF = 44.50; precision, *F*(1.75, 92.46) = 8.25, *p* < .001, η_p_^2^ = .14, BF = 54.44. Further comparisons indicated that error was lower and precision higher for high-priority relative to both no-priority items (error, *p* = .002, *d* = .45, BF = 17.55; precision, *p* = .006, *d* = .39, BF = 5.42) and low-priority items (error, *p* = .001, *d* = .46, BF = 22.20; precision, *p* = .001, *d* = .46, BF = 22.20). There was no significant difference between no-priority and low-priority items on either measure (error, *p* = .064, *d* = .26, BF = .78; precision, *p* = .16, *d* = .20, BF = .39).

### Discussion

The results show a pronounced recency effect, consistent with previous findings using a continuous measure (Gorgoraptis et al., 2011) and our own extensive data using categorical recall (e.g., Allen et al., 2006, 2014; Atkinson et al., 2018; Berry et al., 2018; Hu et al., 2016; Hu et al., 2014). They show also that the effects of prioritization by value when recalling sequentially presented items are broadly similar to those reported by Atkinson et al. (2022) for items presented simultaneously and by Gorgoraptis et al. (2011) for items presented sequentially but with prioritization induced by the probability of an item being tested. The effects of prioritization seen here also fit with those we have reported previously using categorical measures of feature recall for items presented either sequentially (Allen et al., 2021; Atkinson et al., 2018; Hitch et al., 2018; Hu et al., 2014; Hu et al., 2016) or simultaneously (Allen & Ueno, 2018). Taking the results of the various studies together, the effects of prioritization consist of enhanced retention of information assigned high priority at the cost of poorer retention of information allocated lower priority. The two effects tend to cancel one another out in that introducing prioritization has no effect on retention overall.

Thus, there is broad agreement between results obtained using continuous and categorical recall as measures of retention, consistent with the simple view that categorical recall is based on a continuum of feature representation in memory analogous to the sensory continuum thought to underly categorical perception. Our results also suggest similarity between the effects of inducing prioritization by testing items with different probabilities and assigning them different notional rewards while testing them equally often. While we agree with the idea of a greater automatic basis for prioritization based on learned probabilities (Atkinson et al., 2018), the similarity with value-based effects suggests a common mechanism, which, like Atkinson et al., (2022), we attribute to priorities in encoding and attentionally refreshing different items.

## Experiment 2

Having shown further parallels between the effects of recency and prioritization on both continuous and categorical measures of retention, we turn now to the question whether there are also parallel effects of suffix interference. To address this, we repeated two of the prioritization conditions of Experiment 1 (SP1-Priority vs. No Priority), and on an unpredictable 50% of trials followed sequence presentation with a stimulus suffix, a potentially interfering item that did not have to be recalled. To maximize the amount of interference the suffix was ‘plausible’ (i.e., its color was selected from the same pool as study items; Hu et al., 2014; Ueno, Allen, et al., 2011; Ueno, Mate, et al., 2011). In our previous research using categorical recall we find that when all items have equal priority a suffix has its greatest disrupting effect on the most recent item and none on the first. However, prioritizing the first item not only boosts its recall but renders it susceptible to suffix interference (Hu et al., 2014). These interference effects are associated with a tendency to make binding errors based on the suffix. We have interpreted this set of observations in terms of the suffix tending to enter the focus of attention, which is normally occupied by the most recently presented item and is also occupied during the attentional refreshing of a high-priority item (Hitch et al., 2020).

### Method

#### Participants

Power analysis was conducted based on data from Hu et al., 2014, which found a moderate effect size (Cohen’s *d* = .756) of suffix interference. According to G*Power (Faul et al., 2007), 25 participants would be sufficient to achieve a power of 0.95 at alpha level of 0.05. Thirty-nine students (aged 18–32 years, mean age 21 years, 30 females, 10 males) from the University of York and the Northeast Normal University of China were tested individually and were paid or given course credit for participation. All participants reported having normal color vision.

#### Design and procedure

Stimuli and procedure were taken from Experiment 1 except that (1) there were two baseline (No Priority) blocks with one SP1-Priority block implemented in between, and (2) a to-be-ignored suffix was presented on a random 50% of trials. Each baseline block consisted of 12 practice trials and 60 experimental trials. The SP1-Priority block consisted of 12 practice trials and 120 experimental trials (40 for each serial position).

On suffix trials, a 500-ms suffix was presented at the center of the invisible circle 500 ms after the offset of the last study item (see Fig. 1b). The suffix was selected from the same pool as study items, subject to the constraint that neither its color nor orientation matched any of them. Participants were instructed to ignore the suffix but not close their eyes or look away from it. On control trials a blank screen was presented for 500 ms instead of a suffix. As a result of pilot work both the suffix and the blank screen were accompanied by an auditory beep to help participants discriminate the suffix from study items.

### Results

Comparison of the two no-priority trial blocks indicated no significant difference for error (Block 1 = .74, *SE* = .03; Block 2 = .70, *SE* = .04), *t*(38) = 1.55, *p* = .13, *d* = .25, BF = .52. Precision did somewhat improve between blocks (Block 1 = .52, *SE* = .04; Block 2 = .62, *SE* = .06), *t*(38) = 2.29, *p* = .028, *d* = .37, BF = 1.76, though in each case there is anecdotal Bayes factor support. All subsequent analysis combines the data from the two no-priority blocks.

Mean absolute error and precision are displayed in Fig. 3A and B respectively, as a function of priority condition, suffix, and serial position. A 2 × 2 × 3 (priority condition by suffix by serial position) repeated-measures ANOVA was carried out on each response outcome. Firstly, this indicated a significant effect of priority condition: error, *F*(1, 38) = 5.30, *p* = .027, η_p_^2^ = .12, BF = 1.26; precision, *F*(1, 38) = 10.90, *p* = .002, η_p_^2^ = .22, BF = 11.64, with better overall accuracy during the SP1-Priority condition compared with No Priority. There was a significant effect of suffix: error, *F*(1, 38) = 28.21, *p* < .001, η_p_^2^ = .43, BF > 1,000; precision, *F*(1, 38) = 26.29, *p* < .001, η_p_^2^ = .41, BF = 93.95, with lower accuracy when a suffix was presented. There was also an effect of serial position: error, *F*(1.64, 62.42) = 2.95, *p* = .069, η_p_^2^ = .07, BF = 4.45; precision, *F*(1.32, 50.18) = 4.49, *p* = .029, η_p_^2^ = .11, BF = 44.18.

Furthermore, serial position was involved in significant interactions with both priority condition: error, *F*(1.73, 65.61) = 4.73, *p* = .016, η_p_^2^ = .11, BF = 3.00; precision, *F*(1.49, 56.62) = 7.69, *p* = .003, η_p_^2^ = .17, BF = 161.44, and suffix: error, *F*(2, 76) = 9.42, *p* < .001, η_p_^2^ = .20, BF = 7.60; precision, *F*(2, 76) = 4.50, *p* = .017, η_p_^2^ = .11, BF = .72. For priority, comparisons at each serial position indicated better performance at SP1 when prioritizing that position (error, *p* = .004, *d* = .49, BF = 9.16, precision, *p* = .001, *d* = .56, BF = 27.78) but no difference at the later two positions (*p* > .50, *d* < .11, BF < .21, for each measure). The effect of a suffix was significant at Serial Position 3 (error, *p* < .001, *d* = 1.30; BF > 1,000; precision, *p* < .001, *d* = 1.01, BF > 1,000) but not at the first two positions (*p* > .05, *d* < .30, BF < 1, for each measure). Finally, the suffix by priority interaction: error, *F*(1, 38) = 1.45, *p* = .237, η_p_^2^ = .04, BF = .20; precision, *F*(1, 38) = .02, *p* = .88, η_p_^2^ = .00, BF = .14, and the three-way interaction were not supported: error, *F*(2, 76) = 2.26, *p* = .112, η_p_^2^ = .06, BF = .27; precision, *F*(2, 76) = 1.90, *p* = .16, η_p_^2^ = .05, BF = .25.

**Fig. 4:**
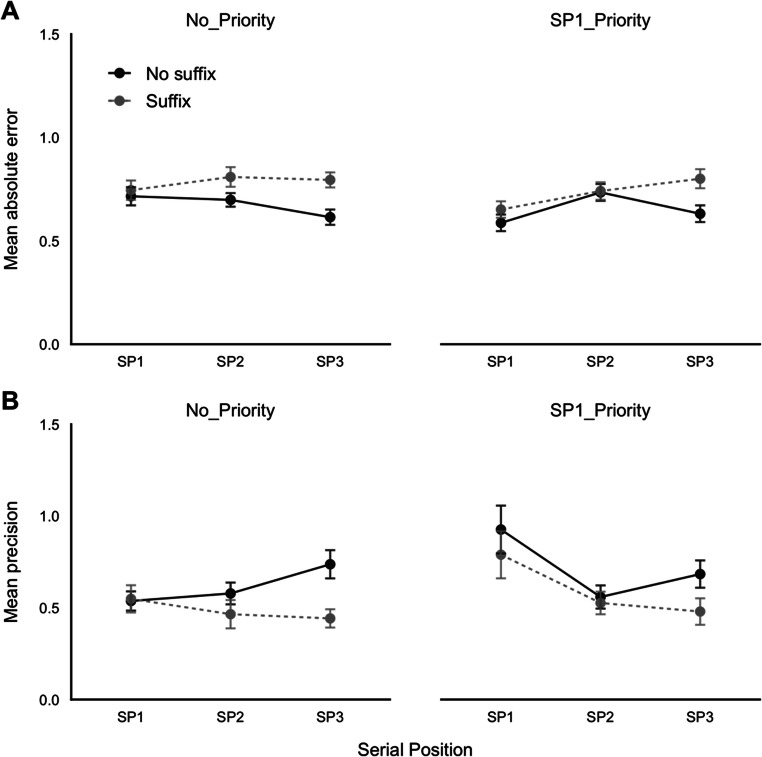
Absolute error (**A**) and precision (**B**), by priority condition, suffix, and serial position. Error bars show *SE*

Although the three-way interaction was not supported, our a priori hypothesis was for a disruptive effect of the suffix at SP1 only when this item was prioritized. In line with this, paired-sample *t* tests on absolute error indicated significant suffix interference at SP1 in the priority condition, *t*(38) = 2.74, *p* = .009, *BF* = 4.39, but not in the no-priority condition, *t*(38) = .44, *p* = .125, *BF* = .23. The precision measure of performance at SP1 showed the same pattern but the critical difference was not significant: priority, *t*(38) = .1.25, *p* = .219, *BF* = .36; no-priority, *t*(38) = .228, *p* = .821, *BF* = .18 Fig. 4.

### Discussion

The effects of serial position, prioritization and their interaction broadly replicated the results of Experiment 1. These consist of a recency effect modified by enhanced recall of the prioritized item. We also observed a disruptive effect of a poststimulus suffix that was greatest for the most recently presented item, in line with our previous findings obtained with categorical recall (Hu et al., 2014).

Some of the other outcomes did not fit so well with previous work. Firstly, in contrast to our findings in Experiment 1 and our previous work using categorical retention (e.g., Allen et al., 2021; Atkinson et al., 2018; Hu et al., 2014), we observed an overall main effect of prioritization. We suggest it would be inappropriate to place weight on this single partial failure to replicate. Secondly, we found no support for the three-way interaction between prioritization, suffix, and serial position found previously in categorical recall (Hu et al., 2014). However, the predicted tendency for the first item to become sensitive to suffix interference when prioritized was observed, albeit as a weak trend and only supported on one of the two outcome measures.

## General discussion

We set out to examine whether visual working memory phenomena described previously using categorical measures of retention look the same on a continuous measure that provides indices of error and precision. We focused on effects of serial position, prioritization, a poststimulus suffix, and the ways they interact, for which we had extensive evidence from our previous work. To the extent these former results generalize, this helps bridge what we perceive as an emerging gap between the two methodologies. It will also encourage the development of more comprehensive theoretical accounts and a deeper analysis of how categorical and continuous feature information in visual working memory are interrelated.

Turning first to serial position, we found a recency effect in both experiments. This parallels the ubiquitous evidence for recency using categorical measures of retention and confirms some previous data for precision (Gorgoraptis et al., 2011). Each of the present experiments also showed that prioritizing one item in a sequence boosts its retention at the expense of items assigned lower priority. The absence of any effect of prioritization on the overall amount of information retrieved in categorical recall (e.g., Atkinson et al., 2018; Hitch et al., 2018; Hu et al., 2018) was replicated in Experiment 1, consistent with prioritization altering the distribution of limited attentional and storage resources in working memory (Hitch et al., 2020). However, as already noted, Experiment 2 here failed to replicate this fully, showing slightly improved overall performance with prioritization than without. We are nevertheless inclined to interpret the overall balance of evidence as showing broadly parallel effects of prioritization on continuous and categorical measures of retention. Independent evidence on the precision of color recall for simultaneously presented stimuli supports this conclusion (Atkinson et al., 2022).

Turning finally to suffix effects, these only partly replicated our previous results for categorical recall. Thus, we did find a disruptive effect on memory for the most recently presented item but the predicted triple interaction involving suffix, prioritization and serial position was present only as a nonsignificant trend. Targeted examination of suffix interference effects on the first item in the sequence indicated significant disruption only when this item was prioritized, though this predicted pattern was present only for the error measure and not for precision. It is possible that the experiment was not sufficiently sensitive to detect the higher order interaction in the ANOVA model. For example, the previous experiments on categorical recall involved four-item sequences (Hu et al., 2014), whereas three-item sequences were used here to ensure adequate levels of performance.

Our overall conclusion is that the present results extend the evidence that visual working memory phenomena look broadly the same whether observed in terms of continuous or categorical measures of retention. One approach towards explaining this is in terms of a simple analogy with categorical perception, which is often described in terms of the application of a discrete decision criterion to a continuum of sensory evidence. In an analogous way, categorical retention can be viewed as resulting from the application of a discrete criterion to a continuous representation of a recent stimulus in visual working memory. This is almost certainly oversimplistic, especially given evidence that continuous and categorical information can make separate contributions to visual working memory (Bae et al., 2015). It does nevertheless suggest a useful starting point for analyzing similarities and, potentially more critically, differences between results obtained with continuous and categorical measures of retention.

To the extent that our previous findings on the retention of categorical information generalize to measures of the precision of recall, the way we have conceptualized working memory (Hitch et al., 2020) requires some modification. This assumes a short-term store subject to rapid forgetting together with a limited capacity current focus of attention and strategic control processes such as attentional refreshing whereby representations in the store can be reactivated by bringing them back one by one into the focus of attention. Up until now we have described the operation and capacities of this system in terms of categorical representations of objects and their constituent features. However, the present results together with wider evidence from studies of precision suggest that object information in visual working memory is underpinned by continuous representations of their constituent features. While we have suggested an analogy with categorical perception as a way to interrelate the retention of feature and item information, this raises interesting further questions such as whether limited attentional resources are allocated over different levels of representation (see e.g., Ma et al., 2014), and as noted earlier, the possibility of a distinct role for item information, even though this may be minor in many cases (Bae et al., 2015).

Our study has focused on methods of prioritization initially implemented prior to stimulus onset and thus being applied during both encoding and maintenance. Indeed, there is some evidence that at least part of the benefit associated with value-based prioritization is derived during the encoding phase (Allen & Atkinson, 2021). Nevertheless, it is useful to acknowledge research that has employed methods of directing attention poststimulus, during working memory maintenance. In the retro-cueing methodology, previously presented items are visually cued during retention, with the cue normally being highly predictive of which item will be tested (Souza & Oberauer, 2016, for a review). It has been suggested that retro-cued items are protected from visual interference (Makovski et al., 2008; Shepardson et al., 2018; Souza et al., 2016) and have a ‘head-start’ preparation for the response probe (Niklaus et al., 2019; Shepardson et al., 2018; Souza et al., 2016). Alternatively, the directed refreshing method of Souza and colleagues (e.g., Atkinson et al., 2022; Souza et al., 2015; Souza et al., 2018) involves cueing the participant to think of (i.e., refresh) certain items being held in working memory. In both these contexts, evidence for effects on precision are quite mixed, with more consistent impacts typically found for probability of target retrieval rather than on precision itself.

Caution should be taken when drawing any firm parallels between these methods of retroactively applied prioritization and that employed in the present study. The validity with which a cue denotes the test item appears to be important in determining patterns of findings (e.g., Gunseli et al., 2015), and indeed probe validity and value have been shown to have additive effects (Atkinson et al., 2018). Processes applied at encoding are also likely to be important (Allen & Atkinson, 2021). Nevertheless, the observation in the current study and in Atkinson et al. (2022) that value-based prioritization impacts on precision contrasts with the less reliable effects on precision seen in retrospective paradigms. This might reflect the additional benefits of prioritizing during encoding, though more systematic work is required to better understand the differences between methods of directing attention.

The present investigation has also brought methodological issues to our attention that merit wider appreciation and discussion. Superficially, one could take the present results as suggesting it does not matter a great deal whether visual working memory is studied in terms of the precision of retained perceptual features or categories. However, this would be to skate over the contrasting strengths and weaknesses of the two approaches and, importantly, limits on what each can tell us. Considering firstly the use of continuous measures, it would seem the main advantage is to allow a much more fine-grained analysis of the information that is held in visual working memory. Such measures are also potentially more powerful than categorical measures for the simple reason they contain more information. They could also be claimed to have the advantage of permitting the application of stochastic models capable of separating out different components of performance. For example, such models can be used to identify binding errors where recollection is based on a different item from the one probed (e.g., Bays et al., 2011). However, a limitation we encountered in the present investigation is that fitting stochastic models involves collecting large amounts of data and their usefulness is limited when it comes to answering questions about interactions. This is particularly important when, as here, we were interested in interaction effects. Applying such models also requires selection between a range of variants, each with different assumptions and parameters, and implementation of an inappropriate or inaccurate model can lead to invalid conclusions (Hardman et al., 2017; Ricker et al., 2022). A different kind of limitation derives from the adjustment procedures used to assess the precision of recall, where there may be potential problems. One is that responses are likely to be influenced by perceptual factors such as the starting point for the adjustment which may be important in psychophysical judgments. Another is that the process of adjustment involves extended interaction with perceptual input that may tend to over-write the information of interest in working memory. Our pilot work for the present investigation suggests overwriting can be substantial as we found we had to reduce sequence length below what had been intended to avoid floor effects. A further possible limitation of analyzing retention in terms of precision is that current models typically assume perceptual dimensions scale linearly and in so doing fail to consider the potentially important role of psychophysical scaling (Schurgin et al., 2020).

In contrast, categorical measures of retention have several useful features despite not allowing the fine-grained analysis provided by continuous tasks. One is that valuable information can be obtained rapidly in experiments that are short relative to the large amounts of data collection required to apply stochastic models. This allows research to proceed more efficiently, without overly long experimental sessions, and limits time spent exploring blind alleys. Another desirable feature is that based on our experience here, interactions between experimental variables can be more readily detected using categorical measures. Yet another is that components of retention such as binding errors can be readily analysed using categorical measures whereas this depends on model fitting and its assumptions in the case of precision. Last but by no means least, categorical measures are widely used to study working memory across a range of qualitatively different domains such as those involving language and speech whereas continuous measures have, so far, more specialized applicability. This is not necessarily a problem, but there is a danger of work on precision developing in increasing isolation from research on working memory more generally.

None of the above should be viewed as arguing for any inherent superiority in using categorical rather than continuous measures to investigate visual working memory. Perhaps most obviously, it is difficult to see how the categorical approach could be applied to the retention of features that are intrinsically continuous such as rotational orientation as studied here. Indeed, Ricker et al. (2022) have recently suggested that the nature of the relationship between categorical and continuous forms of representation may change across paradigms (e.g., between memory for color, shape, and orientation). Research should continue to explore the extent to which outcomes converge across different measures. Another consideration when using categorical measures to investigate visual working memory is the potential for contamination from verbal recoding and subvocal rehearsal strategies. This may also be a factor in studies of precision (e.g., Souza & Skóra, 2017), though possibly to a lesser extent. It is sufficiently important that attempts to minimize such contamination by requiring articulatory suppression are commonly made regardless of the way retention is assessed.

Taken together, our considerations reinforce the need to continue to carry out further complementary studies of visual working memory using both continuous and discrete measures. Better understanding of the strengths and limitations of the two approaches will inform how best to use them, which seems crucial for investigating the way visual working memory handles information at different levels of representation, and more generally working memory as a multicomponent system. Whereas systematic comparisons between them are clearly necessary for full and detailed exploration of how the system operates, we suggest that categorical measures alone can provide an efficient way of gathering information about its broader aspects.
